# Demand Forecasting Approaches Based on Associated Relationships for Multiple Products

**DOI:** 10.3390/e21100974

**Published:** 2019-10-05

**Authors:** Ming Lei, Shalang Li, Shasha Yu

**Affiliations:** 1 Guanghua School of Management, Peking University, Beijing 100871, China; 2 Penghua Fund Management Co., Ltd., Shenzhen 518048, China; lishalang_leee@pku.edu.cn

**Keywords:** demand forecasting, multiple products, granger causality, correlation, inventory performance

## Abstract

As product variety is an important feature for modern enterprises, multi-product demand forecasting is essential to support order decision-making and inventory management. However, these well-established forecasting approaches for multi-dimensional time series, such as Vector Autoregression (VAR) or dynamic factor model (DFM), all cannot deal very well with time series with high or ultra-high dimensionality, especially when the time series are short. Considering that besides the demand trends in historical data, that of associated products (including highly correlated ones or ones having significantly causality) can also provide rich information for prediction, we propose new forecasting approaches for multiple products in this study. The demand of associated products is treated as predictors to add in AR model to improve its prediction accuracy. If there are many time series associated with the object, we introduce two schemes to simplify variables to avoid over-fitting. Then procurement data from a grid company in China is applied to test forecasting performance of the proposed approaches. The empirical results reveal that compared with four conventional models, namely single exponential smoothing (SES), autoregression (AR), VAR and DFM respectively, the new approaches perform better in terms of forecasting errors and inventory simulation performance. They can provide more effective guidance for actual operational activities.

## 1. Introduction

Demand forecasting, a prerequisite for inventory decision-making, plays a vital role in supply chain management. How to improve prediction accuracy has always been the focus of academic circles and enterprises. With the increasingly fierce competition in business, product variety has become an important feature of modern enterprises, which can contribute to meet diverse needs of customers and occupy more market segments [1]. However, many products, where ‘many’ means hundreds or thousands, bring about a new challenge to demand forecasting. Traditional time series algorithms cannot well adapt to the complex high- or even ultra-high dimensionality, resulting in inferior predictive effectiveness in multi-product scenarios. 

It is worth noting that the demand of multiple products is not completely isolated, but rather complex relationships exist between them. According to the relevant literature, there are two common association relationships between different products: correlation and Granger causality. For example, the demand for complementary products is highly correlated, having contemporaneous influence with each other [2]. Materials used in engineering projects have a clear sequence, so Granger causality exists in their demand [3]. Obviously, capturing and making full use of such potential information can be helpful to obtain more accurate prediction results. What’s more, when the time series are short, historic trend cannot provide enough information for future demand. Associated relationships can make up for the defects and reduce the bias of prediction. However, as far as we know, there is currently little research taking into account association relationships between products in demand forecasting. In this paper, we incorporate associated relationships among products into the forecasting framework to construct a more accurate prediction approach. 

In previous literature, there are mainly three branches of forecasting models for multi-dimensional time series. The first one is a series of statistical methods, represented by the AIRMA model and its extended versions, including VARMA, VARs, BVAR, etc. [4,5,6,7,8,9,10,11,12,13]. They treat multi-dimensional time series as an endogenous system. Target variables are regressed by lag items of all series, considering their relations generally. With the development of econometrics, VARs with different settings are widely applied. For example, [8] proposed five types of VAR and utilized industrial production data from OECD countries to test their forecasting effect. The key defect of VAR is that the number of estimated parameters increases exponentially along with the increase in dimensions. For high-dimensional time series, it is easy to cause overfitting, weakening the prediction ability outside the original sample. Some scholars have assumed that estimated parameters obey a specific prior distribution to reduce their number, i.e., BVAR, applied in macro-economic forecasting [14,15,16,17], market share forecasting [12] and business forecasting [10]. Some other scholars incorporated some unmodeled predictors from exogenous variables to improve original regression models. For example, [18] integrated intra- and inter-category promotional information to construct multistage LASSO regression to forecast the demand of 3222 products. The results are significantly better than the model only using endogenous variables. Unfortunately, these methods can alleviate but not completely solve the problem of overfitting. Accurate results can be obtained only when the time series is long enough.

The second strand of research concentrates on processing high-dimensional time series through the method of dimension reduction, represented by dynamic factor model (DFM). [19] holds the belief that a small number of latent factors are able to interpret fluctuations of observed macroeconomic indices. As long as these potential factors can be portrayed accurately, the task of forecasting is simplified substantially and precise results are achievable. There are many algorithms for the estimation of dynamic factors, including maximum likelihood [19,20,21,22], principle component analysis (PCA) [23,24,25,26,27,28,29,30,31,32], and data shrinking methods [33,34]. As for prediction accuracy, [35] collected relevant literature and confirmed that DFM performs better than single time series prediction models through a meta-analysis method. Reference [36] pointed out that a simple AR model may be better than a DFM model when there is a large structural change in the data. Compared with other high-dimensional time series models like shrinkage forecasts, FDM is also superior [37]. What’s more, [38] introduced U.S. macroeconomic data to compare two forms of DFM estimation methods [39,40]. The results demonstrated that their forecasting precision is not significantly different. However, DFM has the obstacle to tackle sophisticated high-dimensional time series, due to the existence of some isolated series, and the same is true for ultra-high dimensional time series. More specifically, some unique information may be skipped in the process of selecting a limited number of factors, leading to inefficient estimates. If add more dynamic factors, it will fall into the over parameterized problem again.

As the development of artificial intelligence, various machine learning models have been widely used in the area of forecasting, including neural network [41,42,43], support vector machine [44,45,46], nearest neighbor regression [47,48], and so on. They are serious contenders to classical statistical models and form a vital research branch. Different from statistical models, these models construct the dependency between historic data and future values through a black-box and nonlinear process. Reference [49] compared eight types of machine learning models, finding that their rank is unambiguous and does not change much with different features of time series. Reference [50] tested the accuracy of some popular machine learning models. The results demonstrated that their performances were inferior to eight traditional statistical ones. In addition, [50] points out that machine learning models need to become more accurate, reduce their computation load, as well as be less of a black box. Therefore, in this paper, we will continue to optimize the statistics models by associated relationships to get higher accuracy, instead of machine learning models.

By summarizing previous literature related to multi-dimensional time series analysis, we find that these methods all fail to deal with the situation where product dimension is large but time dimension is small. Based on this situation, we innovatively construct an improved forecasting model for the target variable based on its precedent values and endogenous predictors selected from associated relationships. In some scenarios, if there are many time series associated with the object, we adopt two feasible schemes to simplify the variable space. Then we conduct an empirical study by using an actual dataset of a Chinese grid company. The results of forecasting errors and inventory simulation show that new approaches are superior to these conventional time series forecasting models, including SES, AR, VAR and FDM. Generally speaking, the proposed methods have three major advantages. Firstly, the number of estimated parameters is simplified significantly, not depending exponentially on the dimensions. Secondly, each variable has a customized forecasting regression, which can describe isolated time series well. Thirdly, it does not necessary to collect extra data to act as exogenous variables. Therefore, one contribution of our work is that the new approaches innovatively incorporate associated relationships into demand forecasting, getting rid of the transitional dependence on historical data, so it can be applied to forecast short time series with large dimensionality, making up for the void of previous algorithms. In addition, we contribute to solving over-fitting problems, providing a new direction for the subsequent research. Besides the above theoretical implications, the study also has important practical significance. Note that life circles of products especially high-tech products are getting shorter and many new products are born due to the acceleration of technological innovation. Demand forecasting in terms of limited time points is very common and necessary in actual business activities. Therefore, our new approaches have a wide range of application scenarios and can provide more accurate decision-making basis for practitioners. 

The remainder of this paper is organized as follows: In Section 2, we give a brief description of two conventional forecasting models for multi-dimensional time series, i.e., VAR and DFM, and then present our new forecasting approaches based on correlation and Granger causality, respectively. Section 3 describes the procurement dataset and analyzes the relationships between the demands for purchased products. An empirical study and its results are discussed in Section 4. Finally, we summarize our conclusions in Section 5.

## 2. Forecasting Model and Evaluation 

In this section, first we review two common models used in multivariable forecasting. Then we detail our new approaches, utilizing correlations and Granger causality among products to improve prediction accuracy respectively. Finally, some indices are introduced to evaluate the forecasting performance.

### 2.1. VAR and DFM

VAR and DFM are two conventional models used to forecast demand under multi-product scenarios. Both them have specific limitations, struggling with high (or ultra-high) dimensionality and failing to describe evolutions of short time series. Firstly, we introduce the VAR model. Assume that demand of N products at time t is $x_{t}={\left(x_{1,t},x_{2,t},\ldots ,x_{N,t}\right)}^{\prime }$, t = 1, 2, …, T, where $x_{j,t}$ represents the demand of jth product at time t. The VAR model is as follows:

(1) $$B\left(L\right)x_{t}=\alpha +\varepsilon_{t}$$

where $B\left(L\right)=I_{N}-B_{1}L-B_{2}L^{2}-\cdots -B_{p}L^{p}$ is a matrix polynomial with p lags in total. $B_{j}$ is a N×N parameter matrix of jth lag and L is the lag operator calculated by $L^{j}x_{t}=x_{t-j}$. α is a N×1 constant vector, and $\varepsilon_{t}$ is a N×1 vector of white noisy process, without contemporaneous correlation. According to (1), it is obvious that there are total P×N×N free parameters need to be estimated in VAR model. With the increase of the number of products (i.e., N), the parameters increase quadratically. Therefore, only time series have moderate dimensionality, i.e., the length of data point is long enough relative to the number of products, can VAR obtain efficient estimates. 

As for DFM, it extracts some dynamic factors that can explain the most variation of target variables as predictors, turning the curse of dimensionality into a blessing. However, when the number of products is large, there are some isolated products unavoidably. Common factors cannot explain their demand accurately. Keeping the previous assumptions about $x_{t}$, the general DFM model is as follows:

(2) $$x_{t}=\Gamma \left(L\right)f_{t}+\varepsilon_{t},$$

(3) $$\Psi \left(L\right)f_{t}=\eta_{t},$$

where $f_{t}={\left(f_{1,t},f_{2,t},\ldots ,f_{m,t}\right)}^{\prime }$ is a m-dimensional column vector, representing values for m (m<N) unobserved factors at time t. It can supplant the originally large data. $\Gamma \left(L\right)=\Gamma_{0}+\Gamma_{1}L+\Gamma_{2}L^{2}+\cdots +\Gamma_{p}L^{p}$, $\Psi \left(L\right)=I_{m}+\Psi_{1}L+\Psi_{2}L^{2}+\cdots +\Psi_{q}L^{q}$, and the meanings of these parameters are similar to B(L)’s in the previous part. $\varepsilon_{t}$ and $\eta_{t}$ are residuals, satisfying some idiosyncratic assumptions. Equation (3) aims to get predictive values of dynamic factors, then applied in Equation (2). 

We can see that the quality of factors is the key to determine the accuracy of DFM. As mentioned above, there are many methods to extract factors. Among them, PCA is commonly used in forecasting literature [28]. In PCA estimation, assume that $\Gamma \left(L\right)=\Gamma_{0}$, i.e., original time series are only influenced by contemporaneous factors. Because $f_{t}$ and $\varepsilon_{t}$ are uncorrelated at all lags, we can decompose the covariance matrix of $x_{t}$ into two parts:

(4) $$\Sigma_{xx}=\Gamma_{0}\Sigma_{ff}\Gamma_{0}^{\prime }+\Sigma_{\varepsilon \varepsilon }$$

where $\Sigma_{ff}$ and $\Sigma_{\varepsilon \varepsilon }$ are covariance matrices of $f_{t}$ and $\varepsilon_{t}$ respectively. Under the assumptions, the eigenvalues of $\Sigma_{\varepsilon \varepsilon }$ is O(1) and $\Gamma_{0}\Gamma_{0}^{\prime }$ is O(N), the first r eigenvalues of $\Sigma_{xx}$ are O(N) and the remaining eigenvalues are O(1). Therefore, the first m principal components of $x_{t}$ can act as dynamic factors. If $\Gamma_{0}$ is known, the estimator of $f_{t}$ can be calculated by OLS directly, i.e., ${\hat{f}}_{t}={\left(\Gamma_{0}{\Gamma_{0}}^{\prime }\right)}^{-1}{\Gamma_{0}}^{\prime }x_{t}$. However, $\Gamma_{0}$ is usually unknown for most cases. Similar to regression, the following optimization equation can estimate $\Gamma_{0}$ and $f_{t}$:

(5) $$\underset{f_{1},f_{2},\ldots ,f_{T,}\Gamma_{0}}{\min}\frac{1}{T}\sum_{t=1}^{T}{\left(x_{t}-\Gamma_{0}f_{t}\right)}^{\prime }\left(x_{t}-\Gamma_{0}f_{t}\right),\;s.t.\;\Gamma_{0}\Gamma_{0}^{\prime }=I_{r}.$$

The first order condition for minimizing (5) with respect to $f_{t}$ shows that ${\hat{f}}_{t}={\left({\hat{\Gamma }}_{0}{{\hat{\Gamma }}_{0}}^{\prime }\right)}^{-1}{{\hat{\Gamma }}_{0}}^{\prime }x_{t}$. By substituting this into the objective function, the results demonstrate that ${\hat{\Gamma }}_{0}$ equals to the first m eigenvectors of ${\hat{\Sigma }}_{xx}$, where ${\hat{\Sigma }}_{xx}=T^{-1}\left(\sum_{t=1}^{T}x_{t}{x_{t}}^{\prime }\right)$. More detailed derivation process can refer to [28]. Correspondingly, ${\hat{f}}_{t}={{\hat{\Gamma }}_{0}}^{\prime }x_{t}$ is the first m principal components of $x_{t}$. It is the final PCA estimator of dynamic factors in DFM. Finally, let $x_{t,forecast}={\hat{\Gamma }}_{0}{\hat{f}}_{t,forecast}$ to get predictive values of the original time series. 

### 2.2. The Forecasting Approach Based on Correlation

Associated relationships between multiple products can provide rich information for demand forecasting. Mining effective predictors from associated time series, instead of all series, will be helpful to eliminate some irrelevant information and reduce the number of parameters significantly. Based on this believe, we propose new approaches based on two typical association relationships, namely correlation and Granger causality, respectively. It is proved that they have higher accuracy and can work well even if a wide range of products only have limited data points in the time dimension.

We start with the forecasting approach based on correlation between products. If two products are highly correlated, their demand has specific interactions in the contemporaneous period. For example, if the demand for a product increases, its complementary products will also see a rise in demand at the same time, while its substitutes will experience a decline. Therefore, we utilize such hidden information to modify forecasting algorithms and get more accurate results. There are mainly three steps in the forecasting approach based on correlation. Firstly, find a proper variable subset for each product in terms of correlated relationships. To be specific, calculate the correlation coefficients between the target one and all other products. Those highly correlated to the targeted one, i.e., whose correlation coefficient is more than a certain threshold, constitute the proper variable subset. If a product does not have highly correlated ones, its proper variable subset is empty. Secondly, run autoregressive model (AR) for each product to get originally predictive values of its demand. AR only depends on past values of a time series to forecast its future evolution, ignoring useful information hidden in other correlated time series. Therefore, the third step is that reconstruct forecasting model for products whose proper variable subset is not empty. We can add these proper variables into AR to get final results. It is worth noting that in some cases one product may have many highly correlated products, i.e., many proper variables. If the time point is not enough, adding too many proper variables results in the over-fitted problem, similar to VAR. For example, in this paper, the training sample only contains 36 time points in total. In terms of the principle that the number of estimated parameters should be less than 1/10 of that of observations, there are no more than 3 parameters in the forecasting model. Since the autoregressive process of original time series occupies at least two parameters, only one predictor based on correlation can be selected. Therefore, we propose two feasible schemes to control the scale of the forecasting model as follows:

Scheme (I): Only select the product having the highest correlation with the object from the proper variable subset as a predictor added in final model.
Scheme (II): Extract the first principle component of all elements in the proper variable subset as a predictor added in the final model.

More formally, Let $X=\left[x_{1},x_{2},\ldots ,x_{T}\right]$ represent time series of demand for all products during the T periods, and then the correlation matrix $\rho_{XX}$ of X is as follows:

(6) $$\rho_{XX}\left(i,j\right)=\frac{cov\left(X_{i.},X_{j.}\right)}{\sqrt{var\left(X_{i.}\right)\times var\left(X_{j.}\right)}}$$

where $X_{i.}$ is the ith row of X, i.e., the sophistic demand series of ith product. According to $\rho_{XX}$, we can pick up products highly correlated to $X_{i.}$, making up for the proper variable subset for ith product. The autoregression $x_{i,t}=\alpha_{i}+\beta_{i,1}x_{i,t-1}+\beta_{i,2}x_{i,t-2}+\cdots +\beta_{i,p}x_{i,t-p}+\varepsilon_{i,t}$ can get originally predictive demand ${\hat{x}}_{i,t}$ for ith product in tth period. p is a lag parameter, determined by the Akaike information criterion (AIC). Based on this, ${\hat{X}}^{\left[i\right]}$ is a matrix, containing original prediction values of all proper variables for ith product. Its rows represent time dimension and columns correspond to products, ranked from left to right in terms of their correlation coefficients with $X_{i.}$ in descending order. Assume that $f_{i}=\left[f_{i,2},f_{i,3},\ldots ,f_{i,T}\right]$ is the effective predictor selected from ${\hat{X}}^{\left[i\right]}$ to improve forecasting. Because it corresponds to prediction values, the first time point is missed. The final model is as follows:

(7) $$x_{i,t}=\alpha_{i}+\beta_{i,1}x_{i,t-1}+\beta_{i,2}x_{i,t-2}+\cdots +\beta_{i,p}x_{i,t-p}+\beta_{i,p+1}f_{i,t}+\varepsilon_{i,t}$$

Scheme (I) suggests that $f_{i}={\hat{X}}_{.1}^{\left[i\right]}$, where ${\hat{X}}_{.1}^{\left[i\right]}$ is the first column of ${\hat{X}}^{\left[i\right]}$, the product having highest correlation with the ith one. According to Scheme (II), $f_{i}$ is the first principle component of ${\hat{X}}^{\left[i\right]}$. The procedure of calculating principle components is as follows: (i) computing the covariance matrix $\Sigma_{\hat{X}}$ of ${\hat{X}}^{\left[i\right]}$, (ii) determining eigenvalues and eigenvectors $\left(\lambda_{1},e_{1}\right)$, $\left(\lambda_{2},e_{2}\right)$, …, $\left(\lambda_{n},e_{n}\right)$ of $\Sigma_{\hat{X}}$, where $\lambda_{1}>\lambda_{2}>\cdots >\lambda_{n}$, (iii) getting the first principle component $f_{i}=e_{1}^{\prime }{\hat{X}}^{\left[i\right]}$.

### 2.3. The Forecasting Approach Based on Granger Causality

If Granger causality exists between two products, it means that historic observations of one product can explain the future demand of another product (there is a time lag between them). This situation often occurs when the procurement of products has a stable sequence, such as material procurement in engineering projects. The idea of the forecasting approach based on Granger causality is similar to the former one based on correlation, also consisting of three steps. Firstly, find the proper variable subset for every product by doing Granger causal relation test. When the *p*-value of Granger test satisfies a critical condition, the corresponding product can join the proper variable subset of the target one. Secondly, run AR for every product to get its originally predictive demand. Finally, select effective predictors from the proper variable subset to reconstruct the forecasting model, if a product’s proper variable subset is not empty. Similarly, there are also two schemes to prevent excessive parameters:

Scheme (I): Only select the product having lowest *p*-value of Granger test with the object from the proper variable subset as a predictor added in final model.
Scheme (II): Extract the first principle component of all elements in the proper variable subset as a predictor added in the final model.

Assume $P_{XX}^{k}$ is the *p*-value matrix of Granger test for X considering k lags, where k is determined by AIC. The rows of $P_{XX}^{k}$ describe Granger results while columns are Granger causes. According to $P_{XX}^{k}$, we can construct the proper variable subset for the ith product, expressed by a matrix $X^{\left[i,k\right]}$. The granger cause with the lowest *p*-value of the ith product arranges in the first column of $X^{\left[i,k\right]}$, and so on. Let $f_{i}^{k}=\left[f_{i,1}^{k},f_{i,2}^{k},\ldots ,f_{i,T}^{k}\right]$ represents the effective predictor extracted from $X^{\left[i,k\right]}$. According to Scheme (I) and Scheme (II), $f_{i}^{k}=X_{1.}^{\left[i,k\right]}$ and $f_{i}^{k}$ is the first principle component of $X^{\left[i,k\right]}$ respectively. The final forecasting model based on Granger causality is as follows:

(8) $$x_{i,t}=\alpha_{i}+\beta_{i,1}x_{i,t-1}+\beta_{i,2}x_{i,t-2}+\cdots +\beta_{i,p}x_{i,t-p}+\varphi_{i,1}f_{i,t-1}^{k}+\varepsilon_{i,t}.$$

### 2.4. The Forecast Accuracy Measures

According to the previous literature, there are two major methods to evaluate the performance for demand forecasting approaches: forecasting errors and inventory performance, from the perspective of forecasting accuracy and actual inventory management, respectively. It is worth noting that the dataset used in this paper has intermittent demand series: the demand of some products is zero in some periods. Therefore, we adopt absolute scaled error (ASE) to measure forecasting errors. It can overcome the drawback of infinities caused by zero division [51]. The formula is as follows:

(9) $$ASE_{t}=\frac{\left|y_{t}-{\hat{y}}_{t}\right|}{\frac{1}{n-1}\sum_{i=1}^{n-1}\left|y_{i+1}-y_{i}\right|}$$

Then mean absolute scaled error is $MASE=mean\left(ASE_{t}\right)$. A forecasting approach with lower MASE means that it is more accurate during the whole forecasting period in general. Therefore, we can compare different approaches according to their values of MASE. In addition, we also apply relative error (RE) to measure the accuracy of forecasters, i.e., calculating ratios of their ASE to that of a baseline model. In this paper, we set simple exponential smoothing (SES) model as the benchmark, which can refer to [52]. For multi-period demand forecasting, the overall judgement of RE is usually based on the form of geometric mean instead of arithmetic mean [51,53]. Geometric mean relative absolute scaled error is expressed as $GMRASE=gmean\left(e_{t}/e^{*}{}_{t}\right)$, where $e^{*}{}_{t}$ is errors of the baseline model.

In fact, optimizing forecasting accuracy aims to provide better guidance for order strategy and inventory management, finally reducing inventory costs and improving managerial efficiency. How forecasting results influence inventory performance is also a concern for scholars and enterprises. A lot of studies assess forecasts by means of inventory simulations [54,55,56,57,58]. Therefore, we also introduce inventory performance to evaluate forecasting approaches. The order-up-to-level policy, commonly used in practice, is adopted to control inventory simulation. We set the inventory review period as one month, consistent with the prediction period. The order-up-to level S is $S=\hat{D}+SS$, where $\hat{D}$ is the predictive demand during the lead time (one month), SS is the safety stock related to the desired service level. At the beginning of each period, check the holding stock H. If H is below S, place an order with the ordering quantity H-S. Otherwise, nothing needs to be done. When face out-of-stocks, the demand will be serviced in the next period. To initialize the simulation system, assume that have full stock at the beginning, i.e., H=S. One index of inventory performance is total inventory costs, consisting of two parts: shortage costs and holding costs, i.e., total inventory costs = unit total cost × (mean inventory per month × a + mean stock-out per month × b) [52]. The cost parameters a and b reflect the trade-off between stock-holding and out-of-stock. b>a means that costs of out stocks are more expensive. When b<a, by contrast, unit stock-holding costs more dollars. In addition, another index is the inventory ratio. A smaller inventory ratio means higher inventory efficiency. It is calculated by the following expression:

$$\frac{mean\;holding\;stock}{mean\;demand}.$$

## 3. Data Description

### 3.1. Data and Pretreatment

In this paper, we obtained a real dataset of material procurement from a large grid company in China over the span from June in 2012 to April in 2016, comprising a total of 47 months. To minimize forecasting errors, we removed trend and seasonal components from the time series, following [38,45]. 

Because it takes at least three years to estimate seasonal components, we treat the first 36 months as a training set and the remaining 11 months as a test set to evaluate the out-of-sample prediction ability of forecasting approaches. After removing trend and seasonal effect, results of the unit root test suggest that all processed variables are stationary, the subsequent forecasting steps can continue. 

Purchased products are mainly infrastructure materials, consisting of cables, transformers, fittings, etc. Note that the demand is intermittent in the dataset. A few products even have zero demand at more than 2/3 of all time points. These products are not suitable to do forecasting and the data have already be cleaned up. Besides, products without procurement in the first 12 months and the last 12 months are also not considered. In total, there are 338 products left. According to product characteristics, they can be aggregated at different levels, forming a hierarchical structure, which is: Family > Category > Subcategory > Product, from the top level to the bottom level. As shown in Table 1, at the most aggregated level, there are two families, namely primary equipment and equipment material respectively, which can be further disaggregated into 15 categories at level 2 and into 59 subcategories at level 3. Besides, it is obvious that the quantity of products and the value of procurement vary significantly within each categories (subcategories). 

### 3.2. Correlation Analysis

In this sub-section, we compute correlations coefficients between products at different aggregated levels to make clear their dependencies structure, supporting the subsequent forecasting. At the top level, primary equipment and equipment material are highly correlated. The correlation coefficient of the two families is 0.7784.

**Figure 1:**
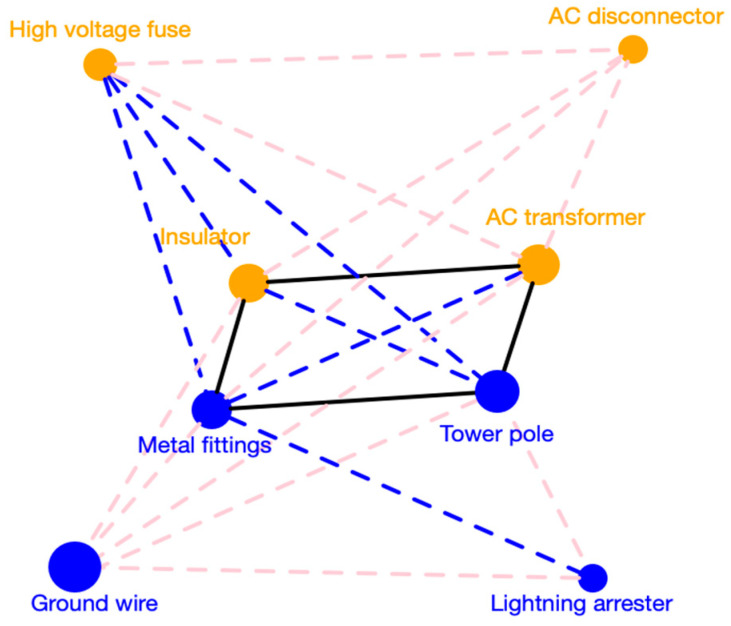
Correlation relationships of categories at level 2.

As for 15 categories, their correlations are shown in Figure 1. A pink dotted line indicates that the correlation value of two linked nodes is in the interval [0.6, 0.7). Similarly, a blue one corresponds to [0.7, 0.8), while a black line means more than 0.8. Seen from Figure 1, there exists high correlations among 8 categories, including AC transformer, insulator, metal fittings, tower pole, AC disconnector, lightning arrester, high-voltage fuse, and wire & ground wire, especially the first four categories. In addition, the correlation network is clustered by a method proposed by [59] and nodes with same colors in Figure 1 represent that they are clustered in a same group. To be specific, AC transformer, AC disconnector, high-voltage fuse and insulator are in G1, while lightning arrester, tower pole, ground wire and metal fittings are in G2, which are mainly consumed in line laying. According to Table 2, a series of centrality indices of metal fittings are almost the biggest in the correlation network, reflecting that its demand has high correlations with demand of all other categories. 

### 3.3. Granger Causality Analysis

We applied the Granger causal relation test to evaluate the relationship between primary equipment and equipment materials. The result shows that they have no statistically significant causality. In other words, demand of primary equipment does not Granger cause that of equipment materials, with *p* = 0.087. Furthermore, the reverse direction is also not significant, with *p* = 0.201.

**Figure 2:**
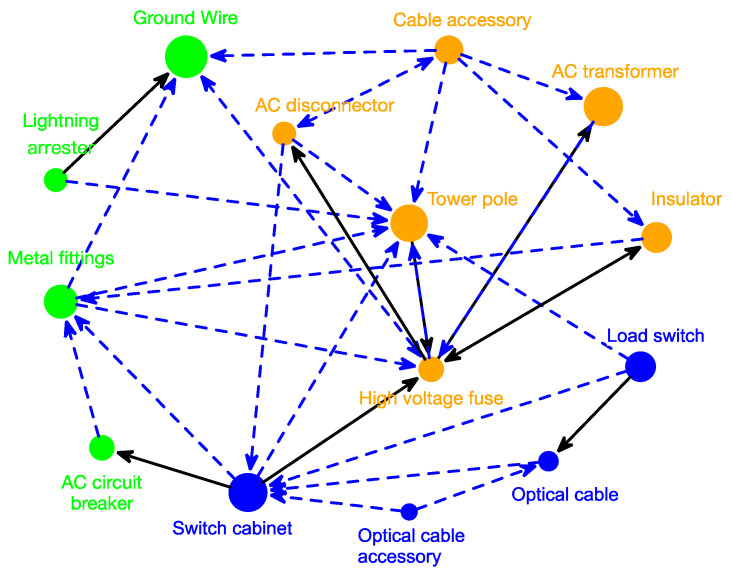
Granger causality relationships of categories at level 2.

In addition, we evaluate the Granger causality relationships among 15 categories at level 2 and visualize the network in Figure 2. Directions of arrows are from Granger causes to Granger results. When the *p*-value of Granger test locates in [0.05, 0.01) and [0.01, 0], the arrow is drawn by a blue dotted line and a black line respectively. Figure 2 shows that the demands of 14 categories have a significant causal influence on each other, except for cable. It is because cables are widely used in power grid construction, not depending on other products. According to the clustering results of causality network, three groups can be found. G1 contains optical cable accessory, optical cable, load switch and switch cabinet, and procurement of first three categories Granger results in that of switch cabinet. G2 consists of cable accessories, AC transformers, insulator, high-voltage fuse, tower pole and AC disconnector, similar to G1 in correlation analysis, existing complex causality relationships. Overall, high-voltage fuse and tower pole locate at core position, which also have causality relationships with out-group products. Lightning arrester, metal fittings, AC circuit breaker and ground wire are clustered in G3, similar to G2 in correlation analysis. The significant direction of causality from the first three categories to wire & ground wire reflects that a growth of their purchase will increase demand for wire & ground wire later. 

To further illustrate Granger causality between categories, we calculate some indices, listed in Table 3. High-voltage fuses and tower poles display the biggest values of in-degree, indicating that their procurement can be greatly explained by lag demand of other categories, while referring to out-degree, high-voltage fuse also ranks first, as well as cable accessory, reflecting that they are strong predictor for follow-up demand of other categories. Besides, switch cabinet, tower pole and high-voltage fuse, with largest centrality indices, are the core nodes in the Granger causality network. what’s more, categories in G1 have closer relationship, demonstrated by their higher clustering coefficients.

## 4. Empirical Analysis

### 4.1. Experimental Setup

In this section, we generate demand forecasting for the above dataset by our new approaches and compare their forecasting performance with four traditional time series models, namely SES, AR, VAR, and DFM, respectively. Except for the enterprise level which only has a single time series, we applied these forecasting approaches on the other four levels of the product hierarchy. Because the number of products varies greatly at the different levels, from several to hundreds, it helps us to investigate whether the new approaches can deal with different data dimensions well. Nevertheless, DFM is not necessary to do forecasting at the family level. VAR ignores the product level and the subcategory level due to a large number of products at the two levels.

An essential step of our new methods is to set up the criteria for constructing proper variable sets, in other words, to define the critical conditions of high correlation and significant Granger causality. If the critical conditions are too strict, most products may not find proper variables, and then their forecasting demand cannot be corrected by association relationships. Conversely, an excessively loose condition will bring too much disturbing information in proper variable sets, even impairing originally forecasting accuracy. Therefore, a rational critical condition is a key point to obtain satisfying prediction results. Considering that in the forecasting approach based on correlation, originally forecasting values of AR are used as explanatory variables, which may further increase uncertainty, a stricter selection criterion is necessary. We set the correlation coefficient at more than 0.513 as the threshold condition preliminarily. The significance level of the critical value is 0.001 in terms of the size of the training sample. As for the Granger relationship, the standard is that the *p*-value of Granger test is no less than 0.1. Based on the above settings, we get proper variable sets and then do final demand forecasting. What’s more, to further investigate the influence of critical conditions on forecasting performance, we set the critical correlation coefficient as 0.6, 0.7, 0.8, 0.9 as well as the critical Granger significant level as 0.05 and 0.01 separately to repeat prediction process. 

Finally, we evaluate the forecasting performance for these models. To begin with we calculate their absolute errors and relative errors in terms of the equations mentioned before. The approach with a smaller average error is considered to be more accurate. Then we introduce *t*-test to verify whether forecasting errors of our new approaches exist statistically significant differences with the baseline model SES. In addition, demand prediction aims to guide subsequent activities including purchase and inventory management. More accurate forecasting may be helpful to avoid high inventory levels or out of stock, reducing the cost loss of enterprises naturally. Therefore, it is common to assess forecasting approaches by simulating the process of inventory management. we also do inventory simulation for 338 products according to forecasting results of different approaches, to compare their performance from the perspective of inventory management. 

### 4.2. Results and Analysis

#### 4.2.1. Forecasting Accuracy Analysis

Table 4 presents mean values of absolute errors and relative errors for the proposed approaches, as well as four conventional models. CI and CII refer to the approach based on correlations adopting scheme (I) and scheme (II) to control model size respectively. Similarly, GI and GII refer to the approach based on Granger causality and the Roman numerals represent different schemes. The brackets indicate the critical conditions to construct proper variable sets. The approach based on correlations requires the correlation coefficient greater than 0.513. As for the approach based on Granger causality, the *p*-value of Granger test should be less than 0.1. 

We can see from Table 4 that for the approaches based on correlations, evaluation results are consistent whether base on MASE or GMRASE. CII performs best among the six models, having a minimum deviation from real demand. Conversely, forecasting accuracy of CI is low, especially at more disaggregated levels. As the dimension of products increase, forecasting errors of CI grows rapidly, even inferior to the original predictive results (AR). This reflects that the forecasting values of the most correlated time series distort the effect of original predictors, leading to lower accuracy, not aligned with our theoretical expectations. However, the first principal component is equivalent to weighted average of all highly correlated time series, which not only contains more effective information but also offsets errors of different correlated series. Therefore, CII can get more accurate results. As for the two types of models based on Granger causality, their accuracy is the same basically at all aggregated levels, superior to that of VAR and DFM. When the product dimension is large, Granger II is more advantageous. 

We set different critical values to investigate their influence on accuracy of models. For the approach based on correlation, we set the critical values separately as 0.6, 0.7, 0.8 and 0.9. Table 5 shows the forecasting errors in each situation. With the increase of the critical value, CI becomes more precise while CII is absolutely opposite, witnessing an upward tendency in errors. However, even if the critical value is equal to 0.9, CI performs still worse than AR, let alone CII. For the approach based on Granger causality, forecasting results in terms of different critical conditions are displayed in Table 6. We can see that forecasting errors of GII is always lower than that of GI. When the critical value equal to 0.01, GII has the highest accuracy. In conclusion, scheme (II) can help the approach based on association relationships to get more accurate forecasting results, better than these conventional models. Besides, the critical value should be set in a rationally high level, to ensure that only highly associated time series can be selected to eliminate irrelevant information and the proper variable subset has enough members too to offset errors. In this way, the approaches based on associated relationships can be the most effective.

To further validate the accuracy of models, we treat the SES model as the benchmark to do *t*-tests for all other models. A negative test statistic means that average ASE of the model is smaller than that of the baseline model, i.e., more accurate than SES model. The smaller the negative t value is, the higher the significance level is. As shown in Table 7, the *t*-test results for absolute errors and relative errors are not consistent. In terms of absolute errors, at the three levels (production, subcategory and category), forecasting errors of GI and GII are all significantly smaller than SES (*p*-value < 0.01), regardless of the critical values. The models based on correlations outperform SES only when adopt scheme (II) to collect predictors. When it comes to relative errors, CI is still significant superior to the benchmark model only at the category level. GI and GII become insignificant in most cases. In conclusion, significance levels of t-value for relative errors are lower remarkably compared to absolute errors. However, CII still has significantly lower errors, performing best among all models.

#### 4.2.2. Inventory Performance Analysis

**Figure 3:**
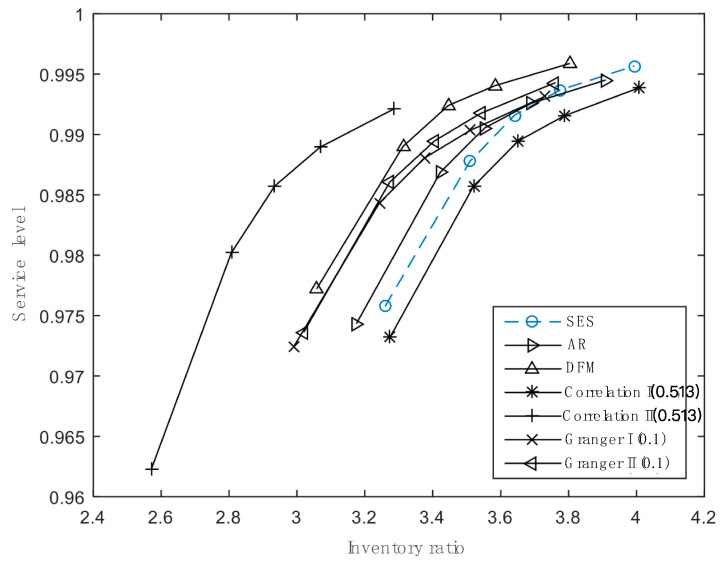
Inventory ratios for different approaches satisfying various service levels.

In our study, we set five desired service levels and three kinds of cost parameters to do inventory simulations for 338 products at the product level. Total inventory costs are shown in Table 8 and Figure 3 illustrates inventory ratios in different scenarios. When satisfying the same service level, CII enjoys the lowest total costs and the inventory ratio and, as expected, CI spends more costs and maintains higher inventory ratios than the original approach (AR) as well as SES and DFM. As for the two approaches based on Granger causality, the results are inconsistent with that of forecasting errors. GI has fewer stock costs and a lower inventory ratio than GII, although GII’s forecast accuracy is higher. In addition, compared with AR and DFM, they need higher inventory levels to realize a certain service level. 

**Figure 4:**
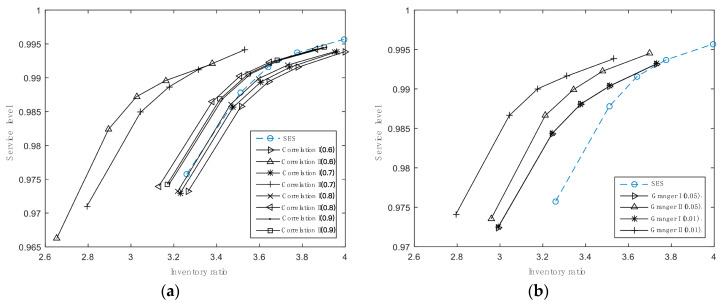
Inventory ratios for approaches with different critical values satisfying various service levels. (**a**) is for the approach based on correlation and (**b**) is for the approach based on Granger causality.

In addition, we also analyze the inventory performance of the proposed approaches satisfying different threshold values. As shown in Table 9, when the critical value rises, total inventory costs creep up if using CII to forecast demand, and the inventory ratios also reach to the top value (in Figure 4a). CI benefits from high critical values, bringing improvement of inventory performance, but never surpasses that of AR.

For approaches based on Granger causality, their inventory performance is displayed in Table 10 and Figure 4b. We can see that total costs and inventory ratios of GI basically remain the same, regardless of the change of the critical value. GII is more sensitive to the critical value in comparison. A stricter condition to select time series existing Granger causality is helpful to optimize inventory performance for a given service level. As a whole, CII with the critical value equal to 0.513 is the most accurate forecasting model, which can help to reduce inventory costs and improve the service level to the most extent, followed by GII with the critical value equal to 0.01. Therefore, by setting rational thresholds and choosing appropriate methods to mine predictors, the approaches based on associated relationships can achieve higher forecasting accuracy, thereby improving inventory performance. It is helpful to provide effective guidance for actual inventory management activities. 

##  5. Conclusions

Along with the trend of technological advance, a rapid increase in product diversity and shortening of product life cycles have become important features for enterprises. This means that practitioners need to use limited historical demand to forecast the future, leading to the failure of traditional forecasting approaches. Fortunately, potential relationships between demand time series of multiple products can provide abundant information to improve forecasting accuracy. Therefore, we proposed an improved approach, whose core idea is utilizing the demand of associated products (including highly correlated ones or ones having significantly causality) as predictors to add in AR model to improve its prediction accuracy. Considering that time series may be short, we introduce two feasible schemes to simplify variables to avoid over-fitting. Then monthly procurement data from a Chinese grid company is used to test the forecasting ability of the proposed approaches, as well as four conventional time series approaches SES, AR, VAR, and FDM. 

We adopt two types of indicators including forecasting errors and inventory performance to compare these approaches. There are three main findings. Firstly, our new approaches can perform better than those conventional approaches, especially when the product dimensionality is large. Among them, CII with the critical value equal to 0.513 has the best forecasting performance, enjoying the lowest forecasting errors, total inventory costs and inventory ratio, followed by GII with the critical value equal to 0.01. Secondly, Scheme (II) is more effective for the approaches based on associated relationships to achieve higher accuracy, compared with Scheme (I) because it can refine more information and set off the errors of different time series. Finally, it is vital to set a rational threshold condition to select proper variables, which can help to get ideal prediction results. The condition should make sure that most objects have enough proper variables to improve prediction and while the irrelative information is also as less as possible. 

However, due to the limitations of the real dataset, we can only pick up one predictor from the associated relationships, so we cannot discuss the influence of the number of selected predictors on the forecasting accuracy. In the future, we can extend our research from the following aspects. Firstly, we can properly test the new approaches through a large wide of diverse datasets, and analyze the most proper number of predictors that should be added in final forecasting models. Secondly, try more methods to dig for predictors from proper variable sets, not limited to PCA. Then compare their prediction performance to find a better one. In addition, “order-up-to level” is the only inventory strategy used to do inventory simulations in this empirical study. Therefore, we can introduce other widely applied strategies into inventory simulations and explore whether the approaches based on associated relationships are appropriate for different inventory strategies.

## Figures and Tables

**Table 1:** Description of hierarchical structure and procurement scale of products.

Family	Category	Sub-Category	Product	Total Purchase	Average Purchase of Subcategory ^1^	Average Purchase of Category ^1^
Primary equipment	AC circuit breaker	1	1	2.65	2.65	2.65
	AC transformer	2	18	102.30	51.15	5.68
	AC disconnector	2	2	2.37	1.19	1.19
	Switch cabinet	3	9	71.25	23.75	7.92
	High-voltage fuse	1	1	4.01	4.01	4.01
	Lightning arrestor	1	1	1.70	1.70	1.70
	Load switch	1	3	11.86	11.86	3.95
Equipment material	Tower pole	2	13	112.90	56.45	8.68
	Wire & ground wire	4	40	140.43	35.11	3.51
	Cable	3	49	272.65	90.88	5.56
	Insulator	6	11	11.29	1.88	1.03
	Metal fittings	24	97	16.75	0.70	0.17
	Cable accessory	5	83	11.01	2.20	0.13
	Optical cable accessory	2	5	0.16	0.08	0.03
	Optical cable	2	2	0.87	0.44	0.44

^1^ Units of average purchase: Million yuan per month.

**Table 2:** Centrality indices of categories in the correlation network.

Category	Cluster	Degree Centrality	Betweeness Centrality	Eigenvector Centrality	PageRank Centrality	Clustering Coefficient
AC transformer	G1	6	0.833	0.125	1.119	0.800
AC disconnector	G1	4	0.000	0.100	0.787	1.000
High-voltage fuse	G1	5	0.250	0.111	0.952	0.900
Insulator	G1	6	0.833	0.125	1.119	0.800
Metal fittings	G2	7	3.000	0.143	1.299	0.667
Tower pole	G2	6	1.417	0.125	1.299	0.733
Ground wire	G2	5	0.667	0.111	0.964	0.800
lightning arrester	G2	3	0.000	0.091	0.631	1.000

**Table 3:** Centrality indices of categories in the causality network.

Category	G	In-Degree	Out-Degree	Betweenes Centrality	Closeness Centrality	Eigenvector Centrality	PageRank Centrality	Clustering Coefficient
Switch cabinet	G1	4	4	52.600	0.056	0.120	1.848	0.179
load switch	G1	0	3	2.667	0.038	0.056	0.768	0.333
Optical cable	G1	2	1	1.000	0.036	0.042	0.808	0.333
Optical cable accessory	G1	0	2	0.000	0.034	0.032	0.575	0.500
AC transfer	G2	2	1	0.400	0.034	0.038	0.543	0.000
High-voltage fuse	G2	6	5	28.967	0.053	0.119	1.578	0.167
Tower pole	G2	7	1	31.067	0.053	0.119	1.583	0.190
Cable accessory	G2	1	5	10.900	0.042	0.074	1.187	0.050
AC disconnector	G2	2	3	3.467	0.045	0.086	0.932	0.417
Insulator	G2	2	2	1.467	0.037	0.059	0.738	0.167
Metal fittings	G3	3	3	19.500	0.050	0.106	1.376	0.300
AC circuit breaker	G3	1	1	0.000	0.037	0.045	0.541	0.500
Lightning arrester	G3	0	2	0.833	0.034	0.037	0.549	0.000
Wire &ground wire	G3	4	1	7.133	0.040	0.067	0.972	0.083

**Table 4:** Forecasting errors of six models at four aggregated levels.

Index	Level	SES ^1^	AR	VAR	DFM	CI (0.513)	CII (0.513)	GI (0.1)	GII (0.1)
MASE	Product	0.7837	0.7895	-	0.7712	0.8102	0.5806	0.7001	0.6953
	Subcategory	0.7376	0.7228	-	0.6820	0.7425	0.5593	0.6749	0.6843
	Category	0.7266	0.6692	0.7985	0.7436	0.6813	0.5412	0.6283	0.6448
	Family	0.8107	0.6697	0.6933	-	0.6693	0.6693	0.6749	0.6749
GMRASE	Product	1	1.1006	-	1.1102	1.1068	0.7918	1.0478	1.0004
	Subcategory	1	1.0310	-	1.0031	1.0560	0.7979	1.0012	1.0151
	Category	1	0.9207	1.1981	1.1029	0.9337	0.7479	0.8687	0.8846
	Family	1	0.8332	0.8514	-	0.8289	0.8289	0.8387	0.8387

^1^ SES is the baseline model when calculate relative errors.

**Table 5:** Forecasting errors of the approaches based on correlation under different critical conditions.

Index	Level	CI	CII	CI	CII	CI	CII	CI	CII
		(0.6)		(0.7)		(0.8)		(0.9)	
**MASE**	Product	0.8095	0.6216	0.7991	0.6926	0.7962	0.7589	0.7933	0.7784
	Subcategory	0.7405	0.5721	0.7400	0.5861	0.7386	0.6112	0.7373	0.6633
	Category	0.6776	0.5346	0.6797	0.5523	0.6651	0.5770	0.6692	0.6692
	Family	0.6693	0.6693	0.6693	0.6693	0.6697	0.6697	0.6697	0.6697
GRMASE	Product	1.1065	0.8516	1.0900	0.9325	1.0990	1.0184	1.1035	1.0760
	Subcategory	1.0528	0.8392	1.0519	0.8403	1.0546	0.8950	1.0499	0.9485
	Category	0.9306	0.7421	0.9353	0.7789	0.9143	0.8094	0.9207	0.9207
	Family	0.8289	0.8289	0.8289	0.8289	0.8332	0.8332	0.8332	0.8332

**Table 6:** Forecasting errors of the approaches based on Granger causality under different critical conditions.

Index	Level	GI	GII	GI	GII
		(0.05)		(0.01)	
	Product	0.7001	0.6602	0.7007	0.6287
MASE	Subcategory	0.6749	0.6607	0.6841	0.6727
	Category	0.6360	0.6572	0.6336	0.6251
	Family	0.6697	0.6697	0.6697	0.6697
GRMASE	Product	1.0478	0.9601	1.0483	0.9159
	Subcategory	1.0012	0.9851	1.0070	0.9768
	Category	0.8814	0.9059	0.8802	0.8686
	Family	0.8332	0.8332	0.8332	0.8332

**Table 7:** *T*-test of forecasting errors for different approaches at three aggregated levels.

Model		ASE			RASE		
		Product	Subcategory	Category	Product	Subcategory	Category
**AR**		0.849	−1.189	−3.301	5.090	0.842	−3.521
VAR		-	-	1.624	-	-	1.780
DFM		−1.392	−2.692	0.398	3.020	0.051	1.154
CI	(0.513)	2.875	0.330	−2.672	6.194	1.564	−2.788
	(0.6)	2.871	0.195	−2.601	6.147	1.471	−2.833
	(0.7)	2.067	0.173	−2.500	5.644	1.458	−2.738
	(0.8)	1.725	0.071	−3.337	5.580	1.404	−3.582
	(0.9)	1.395	−0.021	−3.301	5.317	1.352	−3.521
CII	(0.513)	−14.119	−6.281	−4.799	−11.726	−4.730	−5.256
	(0.6)	−11.002	−5.640	−4.570	−7.693	−3.033	−4.991
	(0.7)	−7.353	−5.008	−3.832	−3.996	−3.271	−4.139
	(0.8)	−2.675	−4.055	−3.221	1.126	−1.915	−3.600
	(0.9)	−0.643	−2.664	−3.301	3.777	−1.016	−3.521
GI	(0.1)	−7.493	−4.178	−3.603	1.405	0.023	−4.114
	(0.05)	−7.493	−4.178	−3.274	1.405	0.023	−3.806
	(0.01)	−7.437	−3.621	−3.450	1.422	0.136	−4.060
GII	(0.1)	−8.197	−3.260	−3.325	0.016	0.202	−3.547
	(0.05)	−10.745	−4.404	−2.282	−1.478	−0.220	−2.564
	(0.01)	−11.906	−3.846	−3.264	−3.566	−0.489	−3.623
Sample size		335	59	15	335	59	15
*p* = 0.1		1.284	1.296	1.341	1.284	1.296	1.341
*p* = 0.05		1.649	1.671	1.753	1.649	1.671	1.753
*p* = 0.01		2.338	2.391	2.602	2.338	2.391	2.602

**Table 8:** Total inventory costs for different approaches considered satisfying various service levels.

GMRASE	Stock Cost Parameters ^1^	SES	AR	DFM	CI (0.513)	CII (0.513)	GI (0.1)	GII (0.1)
0.9	*a* = 0.4, *b* = 0.4	10.96	10.70	10.33	11.02	8.87	10.14	10.22
	*a* = 0.4, *b* = 0.6	11.01	10.76	10.38	11.07	8.94	10.19	10.28
	*a* = 0.4, *b* = 0.8	11.06	10.81	10.43	11.13	9.01	10.25	10.33
0.93	*a* = 0.4, *b* = 0.4	11.83	11.56	11.21	11.87	9.67	11.01	11.10
	*a* = 0.4, *b* = 0.6	11.86	11.59	11.24	11.90	9.71	11.04	11.13
	*a* = 0.4, *b* = 0.8	11.88	11.61	11.26	11.93	9.74	11.07	11.16
0.95	*a* = 0.4, *b* = 0.4	12.29	12.02	11.69	12.33	10.12	11.48	11.57
	*a* = 0.4, *b* = 0.6	12.31	12.04	11.70	12.35	10.14	11.50	11.59
	*a* = 0.4, *b* = 0.8	12.32	12.06	11.71	12.37	10.17	11.52	11.60
0.97	*a* = 0.4, *b* = 0.4	12.77	12.51	12.18	12.82	10.60	11.97	12.06
	*a* = 0.4, *b* = 0.6	12.78	12.52	12.19	12.83	10.62	11.98	12.07
	*a* = 0.4, *b* = 0.8	12.79	12.53	12.20	12.84	10.63	12.00	12.08
0.99	*a* = 0.4, *b* = 0.4	13.57	13.31	12.98	13.61	11.39	12.76	12.85
	*a* = 0.4, *b* = 0.6	13.58	13.32	12.98	13.62	11.40	12.77	12.86
	*a* = 0.4, *b* = 0.8	13.58	13.33	12.99	13.63	11.41	12.78	12.87

^1^ Units of total inventory costs: Million.

**Table 9:** Total inventory costs for the approach based on correlation with different critical values.

Target Service Level	Stock Cost Parameters ^1^	CI	CII	CI	CII	CI	CII	CI	CII
		(0.6)		(0.7)		(0.8)		(0.9)	
0.9	*a* = 0.4, *b* = 0.4	10.99	9.11	10.88	9.53	10.84	10.57	10.72	10.68
	*a* = 0.4, *b* = 0.6	11.04	9.18	10.93	9.59	10.90	10.62	10.77	10.74
	*a* = 0.4, *b* = 0.8	11.10	9.24	10.99	9.65	10.95	10.67	10.82	10.79
0.93	*a* = 0.4, *b* = 0.4	11.85	9.94	11.73	10.40	11.70	11.43	11.58	11.54
	*a* = 0.4, *b* = 0.6	11.87	9.97	11.76	10.43	11.72	11.46	11.60	11.57
	*a* = 0.4, *b* = 0.8	11.90	10.01	11.79	10.45	11.75	11.48	11.63	11.59
0.95	*a* = 0.4, *b* = 0.4	12.30	10.40	12.19	10.86	12.15	11.89	12.04	12.00
	*a* = 0.4, *b* = 0.6	12.32	10.42	12.21	10.88	12.17	11.91	12.05	12.02
	*a* = 0.4, *b* = 0.8	12.34	10.44	12.23	10.90	12.19	11.92	12.07	12.03
0.97	*a* = 0.4, *b* = 0.4	12.79	10.88	12.68	11.35	12.64	12.38	12.52	12.49
	*a* = 0.4, *b* = 0.6	12.81	10.90	12.69	11.36	12.66	12.39	12.54	12.50
	*a* = 0.4, *b* = 0.8	12.82	10.92	12.71	11.38	12.67	12.40	12.55	12.51
0.99	*a* = 0.4, *b* = 0.4	13.59	11.68	13.47	12.14	13.44	13.17	13.32	13.29
	*a* = 0.4, *b* = 0.6	13.60	11.69	13.48	12.15	13.45	13.18	13.33	13.30
	*a* = 0.4, *b* = 0.8	13.61	11.70	13.49	12.16	13.46	13.19	13.34	13.30

^1^ Units of total inventory costs: Million.

**Table 10:** Total inventory costs for the approach based on Granger causality with different critical values.

Target Service Level	Stock Cost Parameters ^1^	GI	GII	GI	GII
		(0.05)		(0.01)	
0.9	*a* = 0.4, *b* = 0.4	10.14	10.03	10.14	9.52
	*a* = 0.4, *b* = 0.6	10.19	10.09	10.20	9.57
	*a* = 0.4, *b* = 0.8	10.25	10.14	10.25	9.63
0.93	*a* = 0.4, *b* = 0.4	11.01	10.91	11.02	10.39
	*a* = 0.4, *b* = 0.6	11.04	10.93	11.05	10.41
	*a* = 0.4, *b* = 0.8	11.07	10.96	11.07	10.44
0.95	*a* = 0.4, *b* = 0.4	11.48	11.38	11.48	10.86
	*a* = 0.4, *b* = 0.6	11.50	11.39	11.50	10.87
	*a* = 0.4, *b* = 0.8	11.52	11.41	11.52	10.89
0.97	*a* = 0.4, *b* = 0.4	11.97	11.86	11.97	11.35
	*a* = 0.4, *b* = 0.6	11.98	11.87	11.98	11.36
	*a* = 0.4, *b* = 0.8	12.00	11.89	12.00	11.37
0.99	*a* = 0.4, *b* = 0.4	12.76	12.66	12.76	12.14
	*a* = 0.4, *b* = 0.6	12.77	12.67	12.77	12.15
	*a* = 0.4, *b* = 0.8	12.78	12.67	12.78	12.16

^1^ Units of total inventory costs: Million.
